# Institutional invisibility versus social acceptance: LGBT students’ perceptions in Brazilian Medical Education

**DOI:** 10.1192/j.eurpsy.2026.11957

**Published:** 2026-07-31

**Authors:** P. F. B. Araújo, V. L. de-Melo-Neto, A. F. C. Ximenes, L. V. de Araújo, B. D. A. A. L. T. de Melo, A. B. D. C. Alves, L. C. Carneiro, M. I. O. de Morais, F. O. Barbosa, E. D. F. Correia, D. B. B. Ferreira

**Affiliations:** 1 Psychiatry, Universidade de Ciências da Saúde de Alagoas; 2 Psychiatry, Universidade Federal de Alagoas, Maceió, Brazil

## Abstract

**Introduction:**

Stigma in academic contexts extends beyond interpersonal discrimination, encompassing the degree of institutional recognition of diversity. Campus climate research highlights that policies, visibility, and support structures for gender and sexual minorities are central to equitable learning environments. In Brazilian medical schools, however, despite broader social progress in LGBT acceptance, formal institutional mechanisms, such as anti-discrimination regulations, diversity centers, or curricular inclusion, remain largely absent. Exploring how LGBT students perceive this gap between social acceptance and institutional invisibility is vital to guide reforms that strengthen equity and mental health.

**Objectives:**

To analyze perceptions of stigma, campus climate, and institutional diversity policies among medical students in a Northeastern Brazilian medical school.

**Methods:**

Cross-sectional study with 142 students (7 gay/lesbian, 3 bisexual, 132 heterosexual; population=593; convenience sample; ≥18 years; informed consent obtained). Instruments: LGBT Campus Climate Scale and Perceived Social Support Scale. Analyses included descriptive statistics, Fisher’s exact test and ANOVA.

**Results:**

LGBT students reported high personal acceptance (78% felt respected by peers) and strong family support (74% agreement). Yet, institutional invisibility prevailed: only 20% of LGBT versus 22.7% of heterosexuals acknowledged anti-discrimination policies. No LGBT student identified resource centers or diversity activities. Moreover, 65% of LGBT respondents stated that faculty rarely addressed diversity in curricula, compared with 42% of heterosexuals (p=0.032).

**Conclusions:**

The coexistence of social acceptance and institutional invisibility highlights a structural gap in medical education. Universities should formalize and disseminate diversity policies, establish support services, and integrate diversity into curricula, fostering equity and protecting student mental health. Findings should be interpreted with caution due to the perception-based design and single-center setting. Future multicenter studies across different Brazilian regions are warranted to capture cultural variations and strengthen the evidence base.

**Disclosure of Interest:**

None Declared

